# Environmental Factors Affecting Thyroid-Stimulating Hormone and Thyroid Hormone Levels

**DOI:** 10.3390/ijms22126521

**Published:** 2021-06-17

**Authors:** Mirjana Babić Leko, Ivana Gunjača, Nikolina Pleić, Tatijana Zemunik

**Affiliations:** Department of Medical Biology, School of Medicine, University of Split, Šoltanska 2, 21000 Split, Croatia; mbabic@mefst.hr (M.B.L.); igunjaca@mefst.hr (I.G.); npleic@mefst.hr (N.P.)

**Keywords:** thyroid hormones, TSH, environmental factors, lifestyle factors, pollutants, diet, chemicals

## Abstract

Thyroid hormones are necessary for the normal functioning of physiological systems. Therefore, knowledge of any factor (whether genetic, environmental or intrinsic) that alters the levels of thyroid-stimulating hormone (TSH) and thyroid hormones is crucial. Genetic factors contribute up to 65% of interindividual variations in TSH and thyroid hormone levels, but many environmental factors can also affect thyroid function. This review discusses studies that have analyzed the impact of environmental factors on TSH and thyroid hormone levels in healthy adults. We included lifestyle factors (smoking, alcohol consumption, diet and exercise) and pollutants (chemicals and heavy metals). Many inconsistencies in the results have been observed between studies, making it difficult to draw a general conclusion about how a particular environmental factor influences TSH and thyroid hormone levels. However, lifestyle factors that showed the clearest association with TSH and thyroid hormones were smoking, body mass index (BMI) and iodine (micronutrient taken from the diet). Smoking mainly led to a decrease in TSH levels and an increase in triiodothyronine (T3) and thyroxine (T4) levels, while BMI levels were positively correlated with TSH and free T3 levels. Excess iodine led to an increase in TSH levels and a decrease in thyroid hormone levels. Among the pollutants analyzed, most studies observed a decrease in thyroid hormone levels after exposure to perchlorate. Future studies should continue to analyze the impact of environmental factors on thyroid function as they could contribute to understanding the complex background of gene–environment interactions underlying the pathology of thyroid diseases.

## 1. Introduction

**Figure 1:**
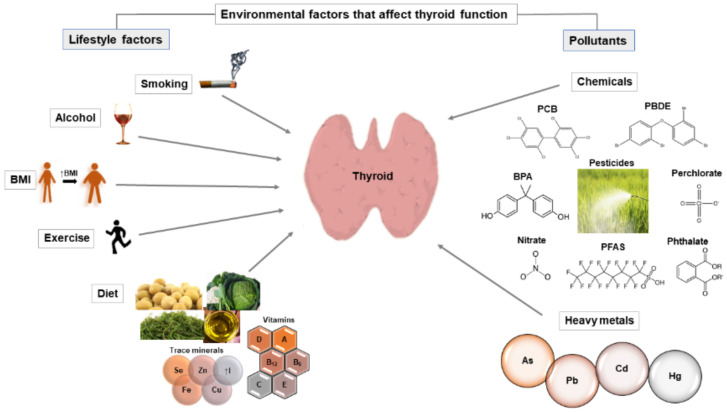
Environmental factors (lifestyle factors and pollutants) that affect thyroid function. As, arsenic; BMI, body mass index; BPA, bisphenol A; Cd, cadmium; Cu, copper; Fe, iron; Hg, mercury; I, iodine; Pb, lead; PBDE, polybrominated diphenyl ether; PCB, polychlorinated biphenyl; PFAS, perfluoroalkyl substance; Se, selenium; Zn, zinc.

Thyroid hormones are crucial for normal development and necessary for the proper functioning of physiological systems. Thyroid hormone synthesis is regulated by feedback mechanisms mediated by the hypothalamus–pituitary–thyroid (HPT) axis. Decreased thyroid hormone levels lead to increased synthesis of hypothalamic thyrotropin-releasing hormone (TRH) which increases the secretion of thyroid-stimulating hormone (TSH) from the anterior pituitary. TSH stimulates the production of thyroid hormones from thyrocytes [1]. Thyroid hormone synthesis requires active iodide uptake through sodium/iodide symporter (NIS), thyroglobulin (Tg) production and Tg iodination by thyroid peroxidase (TPO) enzyme. Thyroid hormones, thyroxine (T4) and triiodothyronine (T3), are released by Tg proteolysis. T4 is released from the thyroid gland in a much larger amount (in a ratio of approximately 14:1) [2]. However, most T4 is converted to T3 in target tissues (by the action of type 1 and type 2 iodothyronine deiodinases (Dio1 and Dio2)) [3]. When secreted in plasma, thyroid hormones are bound to plasma proteins (more than 99.7%) and only a small amount of thyroid hormones are in unbound (free) form (fT4 and fT3). The unbound form of thyroid hormones is biologically active [4]. Variation in the TSH and thyroid hormone levels may indicate that normal thyroid function has been altered. Since the prevalence of thyroid diseases is very high (it is estimated that 12% of the U.S. population will develop a thyroid condition during their lifetime [5]), understanding the mechanisms underlying the variations in TSH and thyroid hormone levels is crucial. Genetic factors account for up to 65% of interindividual variations in TSH and thyroid hormone levels [6,7], but many other factors can also influence thyroid function. Such factors include demographic factors (age and sex [8,9]), intrinsic factors (microbiota [10], stress [11]), usage of medicaments [12] and various environmental factors [13,14,15,16]. The purpose of this review is to provide a comprehensive insight into the literature discussing the impact of environmental factors (such as lifestyle factors and pollutants) on TSH and thyroid hormone levels (Figure 1). Knowledge of any factors that could affect TSH and thyroid hormone levels is especially important for vulnerable groups, such as people with thyroid diseases and pregnant women. However, the focus of this review will be on the general population without thyroid diseases.

## 2. Short Overview of Genetic Factors That Influence TSH and Thyroid Hormone Levels

Twin studies have shown that genetic factors underlie 45–65% of interindividual variations in TSH and thyroid hormone levels [6,7]. Many of these genetic variants have been identified in genome-wide association studies (GWAS) [17,18,19,20]. Genes that contribute to interindividual variations in TSH and thyroid hormone levels are divided into the following groups: genes encoding proteins involved in the synthesis (*TG*, *TPO*, *CAPZB*), metabolism (*AADAT*, *DIO1*, *DIO2*, *DIO3OS*) and transport (*SLC17A4*, *OATP1B1*, *MCT8*) of thyroid hormones; genes for proteins involved in TSH receptor signaling cascade (*TSHR*, *PDE10A*, *PDE8B*, *GNAS*, *ITPK1*); genes encoding growth factors and growth factor binding proteins (*FOXA2*, *IGF2BP2*, *VEGFA*, *IGFBP2/IGFBP5*, *FGF7*, *INSR*, *SASH1*); genes for transcription factors and proteins involved in the development of HPT axis (*SOX9*, *NCOR1*, *FOXE1*, *TTF1/MBIP*, *GLIS3*, *LHX3*, *NFIA*); and genes for proteins with unknown thyroid function (reviewed in [20]). Although major progress has been made in researching the genetic basis of thyroid function, many new potential genetic factors affecting TSH and thyroid hormone levels have yet to be discovered.

## 3. Environmental Factors That Influence TSH and Thyroid Hormone Levels

### 3.1. Lifestyle Factors

#### 3.1.1. Smoking

Most studies investigating the influence of smoking on TSH and thyroid hormone levels have observed a decrease in TSH levels and an increase in T3 and T4 levels in smokers [16,21,22,23]. Large population-based studies have confirmed these results [24,25,26,27,28] (Table 1). In the majority of studies, a decrease in TSH levels was followed by an increase in thyroid hormone levels (Table 1). Recently, in a large cohort of 5766 White North European subjects, Gruppen et al., observed that cigarette smoking leads to a decrease in TSH levels and an increase in fT3 and fT4 levels [16]. Kim et al., even noticed a dose-dependent relationship between cigarette smoking (measured by serum cotinine levels, which is an objective measure of smoke exposure) and TSH levels (study included 4249 participants). They observed that every 10 ng/mL increase in serum cotinine resulted in a 1.4% decrease in TSH levels [28]. It was also observed that TSH levels gradually increased after smoking cessation [27]. The mechanism through which cigarette smoking affects TSH and thyroid hormone levels is still unclear. This is not surprising since there are more than 4000 components in tobacco. One of the proposed mechanisms is that thiocyanate, which is transformed from cyanide in tobacco, inhibits iodide transport and iodine organification (incorporation of iodine into Tg) [29]. Since the transport of iodide is a rate-limiting step in the synthesis of thyroid hormones, exposure to thiocyanate results in decreased thyroid hormone synthesis. However, thiocyanate has been observed to decrease protein-bound T4 levels and consequently increase fT4 levels [30] (which could explain the increase in fT4 levels in smokers). Additionally, several studies have suggested that smoking reduces autoimmune processes in the thyroid gland [24,31], resulting in alterations in TSH and thyroid hormone levels. It has also been suggested that an increase in thyroid hormone levels and a consequent decrease in TSH levels [32] is the result of increased sympathetic nervous activity in smokers [33]. Alarming results were obtained in the study of Filis et al., showing that maternal smoking disrupts fetal thyroid development [34].

#### 3.1.2. Alcohol Consumption

Alcohol has been shown to have a toxic effect on thyroid cells, which is considered to be the cause of decreased thyroid volume in alcoholics [35]. A recent study investigating the influence of alcohol consumption on thyroid hormone levels reported an increase in TSH levels and a decrease in fT3 levels [16]. However, other studies reported conflicting results, with TSH levels being unchanged [36,37,38,39,40] or increased [16,41] in alcoholics, while levels of thyroid hormones were decreased [16,39,40,42], unchanged [36] or increased [38,43] (Table 1). Nevertheless, most of these studies were underpowered, and the study that included the largest number of participants (5766 individuals) detected an increase in TSH levels and a decrease in fT3 levels [16]. Serum Tg levels were increased in patients with chronic alcoholic cirrhosis [44]. Many studies have measured levels of TSH and thyroid hormones during alcohol withdrawal (reviewed in [35]); however, inconsistencies have been reported between studies. Aoun and collaborators even detected a positive correlation between fT3 and alcohol-seeking behaviors in alcoholics [45]. On the other hand, many studies have consistently shown blunted TSH response after TRH stimulation [43,46,47,48]. It has been experimentally proven that chronic ethanol treatment increases TRH levels [49], which could consequently lead to a decrease in pituitary TRH receptors [50] and blunted TSH response after TRH stimulation. Hermann et al., hypothesized that this could be the mechanism by which alcohol leads to alteration in TSH and thyroid hormone levels [51]. They suggested that a decrease in thyroid hormone levels in alcoholics induces an increase in TRH release [51]. Other authors proposed that thyroid dysfunction in alcoholics may be caused by euthyroid sick syndrome (ESS). This syndrome is characterized by decreased levels of T3 and increased levels of thyroid hormone metabolite reverse T3 (rT3). However, the results of many studies did not support this hypothesis (reviewed in [35]). In addition, there is evidence from in vitro and in vivo studies that additional compounds in some alcoholic beverages, such as resveratrol (a natural polyphenol found in red wine) also have a thyroid-disrupting effect (reviewed in [52]).

#### 3.1.3. Body Mass Index

The majority of studies that investigated the influence of body mass index (BMI) on TSH and thyroid hormone levels reported a positive correlation between BMI values and TSH [9,15,23,53,54,55,56] and fT3 levels [57,58,59,60,61] (Table 1). Even high maternal BMI has been shown to be associated with increased fetal TSH levels and increased fetal thyroid weight [34]. However, the results of studies investigating the association between fT4 and BMI were contradictory. Most studies have not observed an association between fT4 and BMI [54,57,59,62,63], although there are studies that have reported negative [9,55,56,60,61,64,65] and even positive association between fT4 and BMI in the general population [66,67,68] (Table 1). Many studies that investigated the influence of BMI on TSH and thyroid hormone levels involved a large number of participants, so sufficient statistical power was reached in these studies. Although for TSH and fT3, there was some consistency of results between studies, this was not observed for fT4. When only studies with a large number of participants (above 1000) were considered, most studies reported a negative correlation between BMI values and fT4 levels (Table 1). The relationship between thyroid hormone levels and weight is well understood in autoimmune disorders. Hyperthyroidism is accompanied by weight loss while hypothyroidism is associated with weight gain [69]. However, the reason for variation in TSH and thyroid hormone levels in euthyroid individuals after an increase in their BMI is not very well understood. Several hypotheses have been proposed. Adipose tissue secretes the hormone leptin which is also involved in the production of hypothalamic TRH (and consequently the production of pituitary TSH) [70]. There is a positive correlation between leptin levels and BMI [71], so this could be a good explanation for why TSH levels increase with increasing BMI. However, some authors think that changes in TSH levels and levels of thyroid hormones are the cause, not the consequence, of an increase in BMI. They propose that lower thyroid function can lead to obesity, probably as a result of a lower metabolic rate [53]. In fact, thyroid hormones have even been used to treat obesity in the past [72], although due to numerous side effects, this weight loss method has been discarded. It has also been speculated that the increase in TSH levels in obesity is a consequence of hormone resistance [73]. This hypothesis could explain why both TSH and T3 levels are increased in obesity. Since T3 receptors are reduced in obesity [74], this could lead to decreased negative feedback between TSH and thyroid hormones and consequently an increase in both TSH and T3 levels. It has also been hypothesized that alterations in TSH and thyroid hormone levels in obesity are due to the process of adjustment to weight gain or subclinical hypothyroidism [73].

#### 3.1.4. Diet

In this section, we discuss how diet can alter TSH and thyroid hormone levels. We do not discuss the well-known dietary iodine deficiency considered to be the most common cause of hypothyroidism in the world [75], as iodine deficiency has decreased dramatically due to the salt iodization programs [76]. We discuss other components in diet that can change the levels of TSH and thyroid hormones, such as soy, brassica vegetables, food associated with the development of endemic goiter, beverages (coffee and tea), other food (junk food, seaweed, spices) and micronutrients (vitamins, trace minerals and macrominerals). It is important to point out that cyanogenic glucosides (reviewed in [77]) and flavonoids (reviewed in [78]) found in a wide range of plant-based food can alter TSH and thyroid hormone levels. Although there are many indications that a particular type of food affects thyroid function, an insufficient number of studies on this issue have been conducted so far. The majority of the studies have investigated the influence of soy-based food on TSH and thyroid hormone levels.

##### Soy

The possible antithyroid effect of soy-based food (soy, tofu, edamame, miso and soy milk) has long been of scientific concern. The goitrogenic compounds found in soy are soy isoflavones, a subclass of flavonoids. Isoflavones are also found in red clover and linseeds. Isoflavones inhibit TPO, an enzyme involved in the synthesis of thyroid hormones [79]. Many in vitro [79,80] and in vivo studies [81,82,83,84,85] have shown that soy isoflavones have a negative effect on thyroid function. Human studies have shown that soy-fed infants developed goiter [86,87,88]. A recent meta-analysis of human studies showed that soy supplementation did not affect thyroid hormone levels and only modestly raised TSH levels [14]. Most human studies have not shown the effect of soy food and goitrogenic compounds found in soy on TSH and thyroid hormone levels [89,90,91,92,93,94]. Some studies however have noticed an increase in TSH levels after consuming soy food or soy isoflavones [95,96,97]. Moreover, an increase [97] and a decrease [95,96,98] in fT4 levels and an increase [97] and a decrease [99,100] in fT3 levels after consumption of soy food or soy isoflavones were observed (Table 1). However, the majority of these studies were underpowered, and additional studies with a larger number of participants are needed to elucidate the influence of soy food and soy isoflavones on TSH and thyroid hormone levels. De Souza dos Santos et al., hypothesized that compared to the other species, the bioavailability of flavonoids to the human thyroid gland may be limited (due to less intestinal absorption and greater hepatic metabolism) [78]. This could explain the less deleterious effect of soy isoflavones on thyroid function in humans compared to experimental animals [78]. However, these authors [78] and others [101] pointed out that soy food and soy isoflavones may have a possible negative effect on thyroid in vulnerable groups, such as people with subclinical hypothyroidism, with iodine deficiency (for example during pregnancy) and with thyroid disorders.

##### Brassica Vegetables

There is much evidence that compounds found in brassica vegetables (also known as cruciferous vegetables) can have a goitrogenic effect [102,103]. Brassica vegetables include broccoli, cabbage, cauliflower, rutabaga, choy sum and turnip. Two compounds identified in brassica vegetables with the potential to have a goitrogenic effect are thiocyanate and goitrin [102]. Thiocyanate and goitrin are produced by myrosinase-induced degradation of indole glucosinolates [104] and progoitrin [105], respectively. Goitrin inhibits iodine uptake by the thyroid gland [30,102,103]. Thiocyanate also reduces iodine uptake by the thyroid since thiocyanate is a competitive inhibitor of NIS [106]. However, human studies have shown no effect of brassica vegetables on TSH and thyroid hormone levels so far [107,108,109] (Table 1).

##### Olive Oil

Many experimental studies have shown that olive derivatives, especially olive oil, have a stimulating effect on the thyroid gland (reviewed in [110]). The experimental studies were performed on various animal models: rats [111,112,113,114], chicken [115,116], lambs [117], goats [118] and calves [119]. The mechanism by which olive derivatives and olive oil affect the TSH and thyroid hormone levels is still unclear [110]. To our knowledge, only one study in humans has shown the effect of olive oil consumption on thyroid hormone levels [120]. Zupo et al., showed that people consuming olive oil have lower levels of fT3 and fT4 [120]. They also observed that the Mediterranean diet, rich in olive oil, slightly inhibits the production of fT3 and fT4 without significantly affecting thyroid function [120].

##### Food Associated with the Development of Endemic Goiter

*Millet*. The flavonoid C-glycosylflavone found in the pearl millet (*Pennisetum glaucum*) inhibited 85% of the TPO enzyme [121]. Pearl millet is a staple food for many people in rural areas of Asia and Africa. Animal studies [121,122,123,124] and epidemiological evidence [123,125,126] suggested that pearl millet may contribute to the development of endemic goiter in areas where this nutrient is consumed. Sartelet et al., proposed that flavonoids present in fonio millet (*Digitaria exilis*) (apigenin and luteolin) also have an antithyroid effect [127].

*Cassava*. Consumption of cassava (*Manihot esculenta*) has been reported to have contributed to the development of endemic goiter in tropical areas where its starchy tuberous root is consumed as staple food [128,129,130,131,132,133]. Linamarin, a cyanogenic glucoside, is thought to be metabolized from cassava to thiocyanate [132,134,135], which reduces iodine uptake by the mechanism previously described.

*Bamboo Shoots*. Consumption of bamboo shoots contributes to the development of endemic goiter [136,137]. Cyanogenic glycosides present in bamboo shoots are metabolized to toxic thiocyanate. Additionally, the in vitro study of Sarkar et al., showed that cyanogenic constituents in bamboo shoots generate reactive oxidative species that contribute to oxidative DNA damage and cell cycle disruption. This is followed by the inhibition of regulatory elements that synthesize thyroid hormones [138,139].

##### Other Food

*Seaweeds*. Seaweeds are frequently used in cuisine in Asian countries. They include different types of algae (green, red and brown) that are accumulators of iodine from the ocean. For example, kelps (the largest of the brown seaweeds) are the main accumulators of iodine in the ocean [140]. While iodine deficiency causes hypothyroidism, iodine excess can cause both hyperthyroidism and hypothyroidism [141]. Studies in euthyroid humans have observed an increase in TSH levels after seaweed consumption [142,143,144] (Table 1). However, Noahsen et al., observed a transient 150% increase in TSH levels in euthyroid individuals after consumption of seaweed (while fT4 levels remained unchanged) that returned to normal within three days [145]. Consumption of seaweed also increased urinary iodine excretion [145]. Miyai et al., also observed an increase in TSH levels after consumption of seaweed “Kombu”, but these levels returned to normal after some time [141]. Additionally, our recent study showed that consumption of seafood (different types of fish and squid) leads to an increase in fT4 and fT3 levels [146] (Table 1).

*Junk Food*. There is evidence that intake of non-home-made meals [147] alters thyroid hormone levels. Consumption of such food increases weight (BMI) and insulin levels [148]. The alteration in TSH and thyroid hormone levels after an increase in BMI is discussed in the previous section. An increase in thyroid hormone levels was also observed after consumption of white bread [146] and pasta [147], while consumption of whole-grain bread [146] leads to a decrease in fT4 levels. Additionally, it was showed that consumption of bacon and sausages lead to an increase in fT3 and fT4 levels [146].

*Spices*. There are proofs from animal studies that some spices can alter thyroid hormone levels. Thus, piperine (the main alkaloid found in black pepper) has been shown to reduce thyroid hormone levels in mice [149]. Cinnamon has also been shown to reduce tT3 levels in rats [150].

##### Beverages

*Tea*. Chandra et al., showed that green and black tea extracts have antithyroid potential [151,152]. It has been shown that catechins (flavonoids found in abundance in tea) have a goitrogenic effect in rats [151,153,154]. An in vitro study showed that exposure to catechins affected thyroid hormone synthesizing enzymes, leading to a decrease in the activity of TPO and 5′-deiodinase I [136]. However, Hu et al., pointed out that there was insufficient evidence of a possible antithyroid effect of catechins in humans [155]. Although perchlorate (a chemical that interferes with thyroid hormone production) was detected in tea samples [156,157,158], it was concluded that exposure to perchlorate due to tea consumption was too low to have a negative health outcome [159].

*Coffee*. Evidence indicates that caffeine blocks the absorption of thyroid hormone replacement therapy (levothyroxine) in patients with hypothyroidism [160]. Although these results have suggested a possible interaction of caffeine with the thyroid hormone system, few studies have been conducted to date on this issue. The results of studies on the effect of caffeine on thyroid hormone levels in experimental animals were inconsistent [161,162]; generally, the existence of a transitory effect of caffeine on the thyroid hormone system with a possible tolerance-related outcome was observed. So far, few human studies have been conducted. Spindel et al., observed no effect of coffee consumption on TSH and T3 levels [163], while Friedrich et al., observed a positive association between urinary trigonelline levels (a marker of coffee consumption) and fT4 levels [164]. The concentration of 3,5-T_2_, which is a metabolic product of T4 degradation, was positively associated with trigonelline levels [165]. In addition, 3,5-T_2_ levels were associated with levels of other compounds in plasma that indicate coffee consumption (caffeine, theophylline, paraxanthine and 1-methylxanthine [166]; trigonelline, pyroglutamate and hippurate [167]).

##### Micronutrients (Vitamins, Trace Minerals, Macrominerals)

Many micronutrients have been shown to have an effect on TSH and thyroid hormone levels. Among vitamins, vitamin D has been the most studied. Moreover, many trace minerals have been shown to have an effect on thyroid function, including selenium, zinc, iron, copper and iodine. Many studies have investigated the effect of micronutrients on TSH and thyroid hormone levels (Table 1). However, it is difficult to draw a general conclusion about how certain micronutrient affects thyroid function due to a high degree of variation between results. These inconsistencies between the results are likely due to the majority of the studies involved being underpowered. Additional studies involving larger cohorts should be conducted.

*Vitamin D*. Vitamin D exerts its effect by binding to the vitamin D receptor (VDR) [168]. It is mainly synthesized in the skin when exposed to sunlight (95%), and only a small amount is taken from food (5%) [169]. VDRs are detected in the pituitary gland, and it is thought that in addition to other pituitary hormones [170,171,172], vitamin D also regulates TSH secretion [173]. In addition, VDRs were detected in cultured rat-derived thyrocytes [174]. The decrease in TSH levels present in the higher status of vitamin D is thought to be the result of an increase in thyroid hormone levels (that is the result of the stimulatory effect of vitamin D on thyrocytes) [175]. Many studies have shown an association between vitamin D deficiency and autoimmune thyroid diseases [176,177,178,179,180]. Additionally, a positive correlation between TSH and vitamin D levels was observed in a study including pregnant women, while fT3 and fT4 levels were negatively correlated with vitamin D levels [181]. These authors suggested that pregnant women diagnosed with transient hyperthyroidism should also be tested for possible vitamin D deficiency [181]. Interestingly, Barchetta et al., suggested that vitamin D influences circannual variation in TSH levels and that seasonal variability in TSH levels in euthyroid individuals depends on vitamin D levels [182]. Studies on the association between vitamin D levels and TSH and thyroid hormone levels in euthyroid individuals have generally observed a negative correlation between vitamin D levels and TSH [175,182,183] and thyroid hormone levels [184] (Table 1).

*Other vitamins*. A deficiency of other vitamins such as vitamin A [185,186], vitamin B_12_ [187], vitamin B_6_ [188,189] and vitamin E [190] has also been observed in thyroid diseases. Supplementation with vitamin C and E [191,192], vitamin A [193], vitamin B_12_ [194] and vitamin B_6_ (reviewed in [195]) has been suggested to improve thyroid health. The influence of a deficiency of these vitamins on TSH and thyroid hormone levels in euthyroid individuals has not been well studied.

*Selenium*. Selenium is an essential trace element that is crucial for the normal functioning of many proteins and enzymes [196]. It is taken from food, mainly meat, grains and seafood. The content of selenium in food is determined by its content in the soil. Thus, some regions with low selenium content in the soil use selenium-rich fertilizers to increase the selenium content in the soil and consequently the intake of selenium by the plants [197]. Selenium is important for the functioning of many enzymes (selenoproteins) involved in the synthesis and metabolism of thyroid hormones and protection against oxidative damage (such as iodothyronine deiodinases, thioredoxin reductases and glutathione peroxidases) [196]. In fact, compared to other organs, the thyroid gland has a high concentration of selenium [198]. Many studies have observed selenium deficiency among patients with benign thyroid diseases [196,199,200]. Thus, selenium supplementation is used to treat various autoimmune thyroid diseases (reviewed in [196]). Most studies in healthy individuals have observed an inverse relationship between selenium concentration and fT4 levels [201,202,203,204,205,206,207]. Regarding TSH and fT3, most studies did not observe significant changes in these hormones after selenium intake (Table 1). It is important to point out that in addition to selenium deficiency, selenium excess is also not good for health [208]. Exposure to high levels of selenium can cause selenosis (when selenium intake is above 850 μg/day) [209].

*Zinc*. Zinc is the second most abundant trace element in the human body and has structural, catalytic and regulatory roles [210]. Zinc is found in meat, milk and fish. Zinc is involved in the synthesis of TSH (since it participates in the synthesis of TRH (as part of zinc-dependent enzyme carboxypeptidase that converts pre-TRH to pro-TRH) [211,212]) and the synthesis of thyroid hormones (as a cofactor of Dio1 and Dio2 [213] and also as part of thyroid transcription factor 2 (zinc-finger protein) [214] that is involved in the transcription of Tg and TPO genes). Zinc is also important for the proper functioning of T3 because T3 nuclear receptors contain zinc ions [215]. Significantly lower zinc levels have been reported in patients with hypothyroidism [216], and some studies have shown a beneficial effect of zinc supplementation on thyroid hormone levels (reviewed in [217]). Studies on the association between zinc levels and TSH and thyroid hormone levels in euthyroid individuals mostly observed no association between zinc and TSH levels (Table 1), negative correlation [208,218] or no association [202,217,219,220] between T4 and zinc levels or positive correlation [219,220] or no association [202,208,217,218] between T3 and zinc levels.

*Iron*. Iron is the most abundant trace element in the human body and is crucial for various cellular functions. Red meat, poultry, fish, leafy greens vegetables, lentils and beans are all rich in iron. Iron is involved in the synthesis of thyroid hormones, and its deficiency can alter thyroid hormone levels in several ways: (1) iron deficiency can reduce TPO activity [221]; (2) iron deficiency can increase rT3 deiodination, leading to thyroid hormone metabolism by inactivating pathway [222]; and (3) iron deficiency can lead to inefficient erythropoiesis, consequently causing a decrease in oxygen transport to tissues. Oxygen is crucial for various enzymatic reactions (including thyroid hormone synthesis) [223]. In a study conducted on 42,162 individuals, Wopereis et al., observed a higher chance of anemia in patients with hypothyroidism and hyperthyroidism [224]. In their meta-analysis, Talebi et al., observed decreased iron levels in patients with subclinical hypothyroidism [216]. Consistent with this finding, another study conducted on 1764 pregnant women showed an increase in TSH levels and a decrease in fT4 levels in the iron deficiency group [225]. Although few, studies in euthyroid individuals mainly observed a decrease in thyroid hormone levels [226,227,228] and no change in TSH levels [226,227,228] in patients with anemia or iron deficiency (Table 1).

*Copper*. Copper is crucial for the normal functioning of many body functions and is an important component of many enzymes. It is also involved in the normal functioning of the thyroid gland and the production of thyroid hormones [229]. Reducing copper levels can increase oxidative stress in thyrocytes because copper is a component of superoxide dismutase that protects cells from oxidative stress [230]. It has also been observed that blood copper levels can change according to thyroid function [229]. Although some studies have indicated a link between copper imbalance and benign thyroid diseases [231,232], a recent meta-analysis by Talebi et al., showed that there was no significant difference in the copper levels between hypothyroid patients and healthy controls [216]. Although few, studies in euthyroid individuals have observed a positive correlation between copper levels and T4 [208,229] and tT3 levels [208].

*Iodine excess*. It is well known that iodine deficiency disrupts the normal functioning of the thyroid gland, but, on the other hand, high iodine intake can also cause thyroid problems. Although most healthy people tolerate high iodine intake well, in vulnerable individuals, it can lead to the development of hyperthyroidism and even hypothyroidism (reviewed in [233]). Causes of excess iodine are the consumption of overiodized salt, seaweeds (as already mentioned) [141,144,145], consumption of excess iodine through water and milk and taking diary supplements that contain iodine [234]. Most studies investigating the effect of high iodine intake on TSH and thyroid hormone levels in healthy adults observed an increase in TSH levels and a decrease in the levels of thyroid hormones after excess iodine [141,144,145,235,236,237,238,239,240,241]. Although only a few studies have been conducted, the pattern of TSH and thyroid hormone levels observed in these studies has shown consistency: an increase in TSH levels followed by a decrease in thyroid hormone levels (Table 1). This hormone profile is a characteristic of hypothyroidism.

*Magnesium*. Magnesium is an essential mineral involved in the functioning of more than 300 enzymes, among which are those important for the synthesis of thyroid hormones [242]. Magnesium is absorbed mainly from magnesium-rich food such as leafy greens, nuts, whole grains and seeds. Some studies have shown a link between magnesium imbalance and benign thyroid diseases [243,244]. However, the meta-analysis of Talebi et al., showed that there was no significant difference in magnesium levels between hypothyroid patients and healthy controls [216]. The effect of magnesium deficiency on TSH and thyroid hormone levels in euthyroid individuals has not been well studied.

#### 3.1.5. Exercise

Exercise affects the homeostasis of the body, the regulation of which involves the HPT axis. Thus, alterations in TSH and thyroid hormone levels were observed after exercise. Additionally, thyroid hormones are involved in the normal functioning of skeletal muscles and pulmonary, cardiac and vascular systems whose activity is significantly altered during the exercise [245]. Many studies have measured TSH and thyroid hormone levels after exercise in healthy individuals, but it is difficult to draw any conclusions due to inconsistencies between studies ([246,247,248,249]; Table 1). A recent study involving 2740 healthy individuals observed no changes in TSH and fT4 levels after exercise [250]. Factors contributing to inconsistencies between studies are the physical status of the subjects, the intensity, duration and type of exercise, differences in age and gender among the subjects and even the ambient temperature [245].

**Table 1:** Effect of lifestyle factors on thyroid-stimulating hormone, thyroid hormone and thyroglobulin levels in healthy individuals.

Factor			Effect on Hormone Levels	Number of Participants	Reference
**Smoking**			↓TSH, ↑fT4, ↑fT3	5766	[16]
			↓TSH, 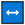 fT4, 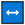 fT3	4585	[146]
			↓TSH, ↑fT4	895	[21]
			↓TSH, 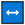 fT4	4357	[28]
			↓TSH	15,181	[27]
			↓TSH, 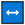 fT4	3404	[251]
			↓TSH	5639	[252]
			↓TSH	4427	[253]
			↓TSH, ↓tT4, ↑Tg	1409	[208]
			↓TSH, ↓tT4, 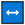 fT4, 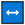 tT3, ↑fT3, ↑Tg	1540	[254]
			↓TSH	1581	[255]
			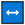 TSH, 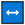 fT4, ↑fT3	931	[256]
			↓TSH, 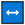 fT4	3399	[26]
			↓TSH, ↓tT4, ↓tT3	237	[257]
			↓TSH, 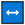 fT4	1853	[64]
			↓TSH, ↑fT4, ↑fT3	7799	[22]
			↓TSH	30,834	[25]
			↓TSH, ↑fT4, ↑fT3	6085	[258]
			↓TSH, 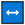 T4	15,592	[24]
			↓TSH, ↑T4	4462	[259]
			↓TSH, 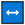 T4, 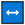 fT4, 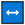 T3, 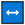 fT3	1154	[260]
			↓TSH, 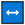 T3	4100	[261]
			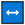 TSH, 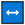 T4, ↑T3	50	[262]
			↓TSH, 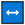 T4, 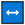 T3, ↑Tg	219	[263]
			↓TSH, ↑T4, 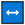 T3	181	[264]
			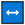 TSH, ↓T4, ↓T3	200	[265]
			↓TSH, 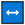 T4, ↑T3, ↓rT3, ↑Tg	441	[266]
**Alcohol consumption**			↑TSH, ↓fT3	5766	[16]
			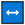 TSH, ↓fT4	549 (men)	[40]
			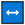 TSH, ↓fT4, ↓fT3	67	[39]
			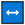 TSH, 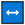 T4, 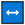 T3	100	[38]
			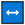 TSH, 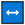 T4, ↑T3	30	
			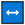 TSH, 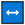 fT4, ↓T4, 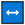 fT3, 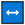 T3	55	[37]
			↑fT4	21	[43]
			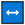 TSH, 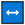 T4, 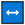 fT4, ↓T3, ↓fT3	70	[36]
			↑TSH, 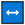 T4, 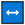 fT4, ↓fT3, ↓T3	80	[41]
			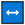 TSH, 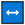 T4, 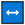 T3, ↑Tg	111	[44]
			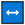 TSH, 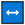 fT4, ↓tT4, ↓tT3	38	[42]
**Increased body mass index**			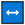 TSH, ↓fT4	90	[267]
			↑TSH (BMI higher than 25.3 kg/m^2^)	11,224	[268]
			↓TSH (BMI lower than 25.3 kg/m^2^)		
			↑TSH	75	[269]
			↑TSH	2789	[15]
			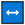 TSH, 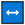 fT4, 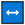 fT3	34	[270]
			↑TSH, ↓fT4, ↑fT3	77,991	[9]
			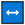 TSH	88	[271]
			↓fT4, ↑fT3, ↑fT3/ fT4	16,975	[65]
			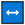 TSH, 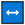 fT4, ↓fT3	36,655 (all subjects)	[272]
			↓fT4	18,746 (men)	
			↑TSH	80	[273]
			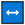 TSH, ↓fT4	7693	[274]
			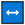 TSH, ↓fT4	1100	[275]
			↑TSH	140	[276]
			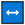 TSH, 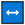 fT4, ↑fT3	940	[277]
			↑TSH, ↓fT4, 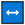 fT3	26,719	[56]
			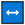 TSH, 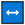 fT4, 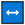 fT3	1275	[278]
			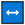 TSH	162	[279]
			↑TSH, ↓fT4	9402	[8]
			↑TSH	800	[280]
			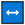 TSH	1097	[281]
			↑TSH, 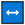 fT4, 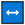 tT4, 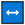 fT3, ↑tT3, ↑Tg	746 (men)	[208]
			↑TSH	1044 (men)	[282]
			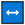 TSH, ↓fT4, ↑fT3, ↑tT3, ↑fT3/ fT4	2315	[60]
			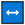 TSH, ↓fT4	6241 (all subjects)	[283]
			↓TSH	2837 (women)	
			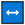 TSH, 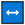 fT4, ↑tT4, 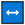 fT3, ↑tT3	736	[67]
			↑TSH	417	[284]
			↑TSH	5918	[285]
			↑TSH, 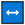 fT4, 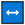 fT3	60	[286]
			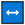 fT4, ↑fT3	865	[287]
			↑TSH, 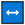 fT4, ↑fT3	3114	[288]
			↑TSH, 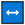 fT4	778	[289]
			↑TSH	1084	[290]
			↑TSH	15,020	[291]
			↑TSH, 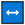 fT4	581	[292]
			↑TSH, 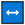 fT4, ↑fT3	520	[59]
			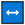 TSH, 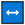 fT4, ↑T3, ↑T3/fT4	275	[293]
			↑TSH, 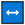 fT4, 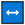 tT3	27,097	[53]
			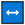 TSH, ↓fT4	44,196	[294]
			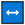 TSH, ↓fT4	1853	[64]
			↑TSH, 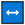 fT4, ↑fT3	152	[242]
			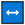 TSH, ↓fT4	1572	[295]
			↑TSH, 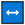 fT4, 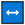 fT3	265	[296]
			↑TSH, 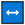 fT4	86	[297]
			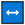 TSH, 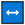 fT4, ↑fT3, ↑fT3/ fT4	201	[57]
			↑TSH	1725	[23]
			↑TSH, 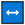 fT4, 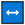 fT3	87	[298]
			↑TSH, ↓fT4, 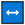 fT3	4082	[55]
**Diet**	Soy food or soy isoflavones		↑TSH, 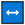 fT4, 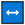 fT3	Meta-analysis	[14]
			↑TSH, ↓fT4, 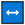 fT3, ↑rT3	400	[96]
			↑TSH, ↓fT4, 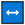 fT3	200	[95]
			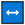 TSH, 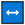 fT4, 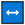 fT3	47	[93]
			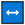 TSH, 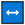 fT4	505	[299]
			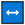 TSH, 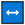 fT4, ↓fT3	43	[100]
			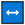 TSH, 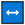 fT4	403	[94]
			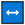 TSH, 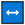 fT4, 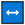 fT3	93	[300]
			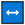 TSH, 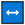 fT4	63	[301]
			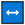 TSH, 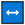 fT4, 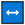 fT3	389	[92]
			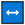 TSH, 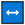 T4	Meta-analysis	[302]
			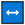 TSH	77	[303]
			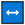 TSH, 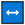 fT4, 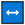 tT4, 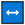 fT3, 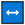 tT3, 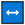 Tg	147	[91]
			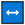 TSH, 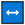 fT4, 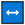 tT4, 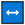 fT3, 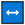 tT3	35	[90]
			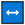 TSH, 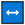 T4, 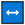 T3, 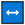 fT4	25	[143]
			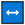 TSH	89	[304]
			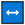 TSH, 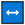 T4, 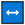 T3	38	[89]
			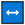 TSH, ↓fT4, 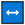 fT3	32	[98]
			↑TSH, ↑T4, ↑T3	73	[97]
			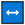 TSH	76	[305]
			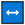 TSH, 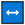 fT4, ↓fT3, 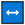 T4, 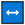 T3	14	[306]
			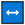 TSH, 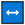 fT4, 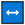 tT4, 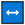 fT3, 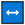 tT3	18	[99]
	Brassica vegetables	Sulforaphane (natural product present in cruciferous vegetables like broccoli)	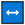 TSH, 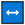 T4, 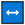 Tg	45	[109]
		Roots of cruciferous plant Lepidium peruvianum Chacon	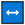 TSH, 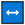 T4, 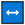 T3	20	[108]
		Brussels sprouts	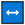 TSH, 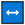 tT4, 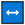 fT4, 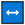 tT3	10	[107]
	Other food	Seaweed	↑TSH, 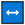 fT3, 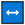 fT4	19	[144]
		Seaweed	↑TSH (returned to normal after several days), 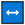 fT4	9	[145]
		Seaweed	↑TSH (returned to normal after several days), ↓fT4, ↓fT3 (returned to normal after several days)	13	[141]
		Seaweed	↑TSH, 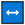 T4, 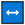 T3, 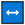 fT4	25	[143]
		Kelp	↑TSH, 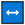 fT4, ↓fT3	36	[142]
		Kelp, vegans vs. omnivores	↑TSH	101	[307]
		Full-fat cheese, cottage cheese, hard cheese	↓fT4	4585	[146]
		Pasta and rice	↑fT4		
		Whole-grain bread	↓fT4		
		White bread	↑fT4		
		White fish, blue fish, dried fish, seafood, squid	↑fT4, ↑fT3		
		Fruit juices, cedevita, nonalcoholic drinks	↓TSH, ↑fT4		
		Pork, beef, eggs	↓fT4		
		Bacon, sausages	↑fT4, ↑fT3		
		Butter, animal fat	↓fT4		
		Canned vegetables, mushrooms	↓fT4, ↓fT3		
		Powder soups, vegetable juices	↑fT4		
		Venison, fish derivates	↓TSH		
		Non-home-made meal	↑T4	100	[147]
		Whole grains, green tea	↓T3		
		Pasta	↑fT4		
	Olive oil	Mediterranean diet	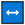 TSH, ↓fT4, ↓fT3	324	[120]
		Olive oil	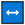 TSH, ↓fT4, ↓fT3		
		Vegetables cooked with olive oil	↑T3	100	[147]
	Food associated with the development of endemic goiter	Cassava	↓T4, ↓T3	20	[308]
	Beverages	Coffee	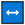 TSH, ↑fT4	9408	[164]
		Coffee	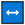 TSH, 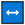 T3	Not reported	[163]
	Micronutrients	↑ Vitamin D	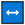 TSH, ↑fT4, 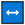 fT3	123	[309]
		↑ Vitamin D	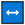 TSH, ↓T4, ↓T3	300	[184]
		↓ Vitamin D	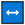 TSH, 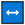 fT4	2006	[310]
		↓ Vitamin D	↑TSH	294	[182]
		↑ Vitamin D	↓TSH	1424	[183]
		↑ Vitamin D	↓TSH	2582	[175]
		↑Selenium	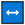 TSH, ↓fT4, 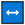 fT3	69	[207]
			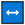 TSH, ↑fT4	184 (women)	[229]
			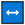 TSH, 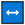 T4, 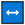 fT4, 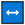 T3, 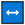 rT3	387	[311]
			↓TSH, ↓fT4, 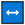 fT3	361	[206]
			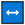 TSH, 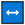 fT4, 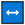 fT3, 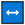 Tg	1383	[208]
			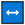 TSH, ↓fT4, ↓fT3	1144	[205]
			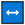 TSH, ↑fT4	140	[312]
			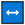 TSH, 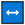 tT4, ↑T3,	28	[313]
			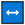 TSH, 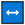 fT4, 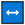 fT3, 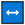 Tg	88	[314]
			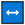 TSH, 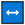 T4, 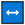 T3	42	[315]
			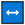 TSH, ↓fT4, 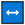 fT3	52	[316]
			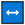 TSH, ↓fT4, 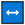 tT4, 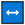 fT3, 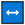 tT3, ↑fT3/fT4	368	[204]
			↑TSH, ↓T3	12	[317]
			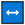 TSH, 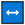 fT4, 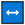 fT3, 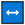 fT3/fT4	44	[219]
			↓T4, 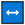 Tg	52	[203]
			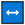 TSH, ↓T4, 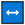 T3, ↑T3/T4	109	[202]
			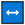 TSH, ↓T4, ↓fT4, 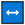 T3, ↓rT3	52	[201]
		↑Zinc	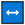 TSH, 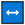 fT4, 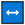 fT3	98	[217]
			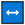 TSH, ↓tT4, ↓fT4, 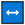 tT3, 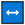 fT3	746 (men)	[208]
			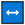 TSH, 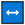 fT4, ↑fT3	64	[220]
			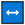 TSH	219	[318]
			↓tT4, 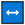 tT3	178	[218]
			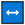 TSH, 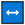 fT4, ↑fT3, ↑fT3/fT4	44	[219]
			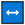 TSH, 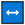 tT4, 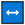 fT4, 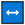 tT3	109	[202]
		Iron deficiency	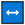 TSH, ↓fT4, ↓fT3	3846	[228]
		Iron deficiency anemia	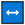 TSH, ↓tT4, 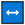 fT4, ↓tT3, 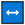 fT3	128	[227]
		Anemia	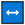 TSH, 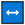 fT4, 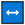 tT4, ↑fT3	50	[226]
		Iron supplements	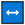 TSH, 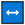 fT4, ↑tT4, ↑tT3, ↓rT3	94	[319]
		Anemia	↓fT4, ↓fT3	20	[320]
		↑Copper	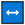 TSH, ↑fT4	417	[229]
			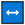 TSH, ↑fT4, ↑tT4, 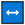 fT3, 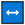 tT3, 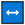 Tg	746 (men)	[208]
			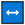 TSH, 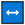 fT4, ↑tT4, 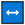 fT3, ↑tT3, 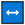 Tg	663 (women)	
		Iodine excess	↑TSH	Meta-analysis	[240]
			↑TSH	78,470	[241]
			↑TSH, 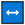 fT4, ↓fT3	854	[239]
			↑TSH, ↓fT4, ↓fT3	236	[321]
			↑TSH, ↓fT4, ↓fT3	256	[238]
			↑TSH, ↓fT4, ↑Tg	10	[237]
			↓T4, 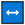 T3	30	[235]
			↑TSH, ↓T4, ↓T3	32	[236]
**Exercise**			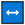 TSH, 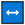 fT4	2470	[250]
			↓TSH, ↑T4, ↑T3	36	[248]
			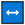 TSH, ↑fT4, ↑fT3	9	[247]
			↑TSH, ↑fT4, ↑T4, ↓fT3, ↓T3	60	[249]
			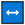 TSH, 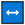 fT4, 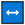 T4, 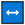 fT3, 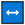 T3	26	[246]
			↑fT4, ↓fT3, ↓T3, ↑rT3	27	[322]
			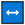 TSH	6	[323]
			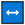 fT4, 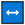 T4, 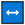 fT3, 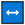 T3, 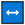 rT3	46	[324]
			↑TSH, ↑fT4	14	[325]
			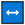 T4, ↑T3, ↑rT3	12	[326]
			↑T4, ↓T3, ↑rT3	4	[327]
			↑TSH	8	[328]

Studies involving pregnant women, infants, children and individuals with a history of thyroid diseases were not included in this table. rT3, reverse triiodothyronine; T3, triiodothyronine; T4, thyroxine; tT3, total T3; tT4, total T4; Tg, thyroglobulin; TSH, thyroid-stimulating hormone.

### 3.2. Pollutants

#### 3.2.1. Chemicals

Many industrial chemicals and pesticides can alter the normal functioning of the thyroid gland. These chemicals are classified as endocrine-disrupting compounds (EDCs) [13]. Because thyroid hormones are crucial in normal brain development [329], any compound that could potentially affect normal thyroid function should be thoroughly investigated. In fact, many studies on the impact of potential EDCs on normal thyroid function have included pregnant women [330,331], infants [332,333] and young children [334,335]. However, these studies are not discussed in this section, which only considers studies including a general healthy population. Although so far many studies have been conducted on the influence of different types of chemicals on TSH and thyroid hormone levels, there is still a high degree of variation between the results. The majority of conducted studies were underpowered, not including a sufficient number of participants (Table 2). In addition, exposure of participants to different subtypes and doses of chemicals could contribute to the differences between the results. Therefore, it is difficult to draw a general conclusion about whether or how a particular chemical affects thyroid function.

##### Polychlorinated Biphenyls and Polybrominated Biphenyls

Polychlorinated biphenyls (PCBs) and polybrominated biphenyls (PBBs) are EDCs. Due to their structural similarities to thyroid hormones, PCBs and PBBs interfere with thyroid hormone signaling [13]. An in vitro study showed that PCBs bind to thyroid hormone receptors [336], and PBBs could affect iodide intake by the thyroid gland [329,337]. PCBs have been widely used as electrical insulating fluids and in carbonless copy paper, inks, paints and other industrial and consumer products. They were banned in the United States in 1979 and again by the Stockholm Convention on Persistent Organic Pollutants in 2001 [338]. However, these chemicals are persistent organic pollutants (POPs) that can accumulate in the environment and body fat and thus can still have detrimental effects on health [13]. PBBs are also POPs and although they are still used as flame retardants (chemicals added to materials used to prevent potential ignition of products), their use is controlled by the 2003 Restriction of Hazardous Substances Directive. Many studies have investigated the influence of PCBs on thyroid hormone levels in healthy adults, but many inconsistencies have been observed between studies. Some studies have not observed the effect of PCBs on TSH [339,340,341,342,343,344,345,346,347,348,349] and thyroid hormone levels [339,340,341,348,350]. However, other studies have shown an increase [351] and a decrease [352,353] in TSH, an increase [347,353] and a decrease [344,346,351,352,354] in T3 and an increase [342,347,349,353] and a decrease [343,346,351,352,354,355,356] in T4 after exposure to PCBs. Although previous studies have investigated the effect of PBBs on the development of thyroid diseases [357,358], we have found only one study investigating the effect of PBBs on TSH and thyroid hormone levels in euthyroid individuals [349].

##### Polybrominated Diphenyl Ethers

Polybrominated diphenyl ethers (PBDEs) are used as flame retardants. Although, to date, the use of most types of PBDEs has been banned or restricted, these chemicals continue to pose a threat to human health because the Stockholm Convention on Persistent Organic Pollutants considers them to be POPs. These chemicals are also EDCs and share structural similarities with T4 [359]. The results of the studies on the influence of PBDEs on TSH and thyroid hormone levels in healthy adults were inconsistent. Some studies did not show an effect of PBDEs on TSH [360,361], T3 [348,360,361] and T4 levels [360,362], while the others observed an increase in TSH [362,363], T3 [344,348,362] and T4 [348,364] levels and a decrease in TSH [364], T3 [364] and T4 [361] levels after PBDE exposure.

##### Bisphenol A

Bisphenol A (BPA) is one of the world’s most commonly used chemical in food packaging, food can lining, toys, tubes, cosmetics, etc. Because BPA is not chemically bound to the material, it can easily diffuse into food or beverages after repeated use, physical manipulation or under high heat [13]. BPA inhibits thyroid hormone synthesis in several ways: it reduces thyroid iodide intake and TPO activity and alters gene expression for proteins involved in thyroid hormone synthesis (reviewed in [365]). In addition, BPA is an antagonist of thyroid hormone receptors [366]. Studies on the influence of BPA on TSH and thyroid hormone levels in healthy adults have yielded inconsistent results. TSH levels were not affected [367,368,369], increased [370] or decreased [371,372,373] after BPA exposure. T3 levels were also unaffected [369,372,373] or increased [368,371] by BPA exposure, while T4 levels were not affected [368,369,370,371,372,373] or decreased [367] by BPA exposure.

##### Phthalates

Phthalates are among the most produced chemicals in the world. They are used as plasticizers and softeners in products such as food packaging, food can lining, toys, tubes, cosmetics, etc. Because phthalates are not chemically bound to the material, they can easily diffuse into food, water and air [374]. In vitro studies have shown that di-(2-ethylhexyl) phthalate (DEHP) has an antagonistic effect on thyroid hormone action [375,376]. In addition, studies in rats have shown that DEHP causes histopathologic changes in the thyroid gland and increases the level of liver enzymes involved in the degradation of thyroid hormones (resulting in a decrease in thyroid hormone levels) [377]. Studies on the influence of phthalates on TSH and thyroid hormone levels in healthy adults have yielded inconsistent results. TSH levels were not affected [372,378,379] or increased [369,372,380] by exposure to phthalates. T3 levels were also not affected [372,379] or decreased [369,372,378] after phthalate exposure, while T4 levels were not affected [372,379,380], increased [379] or decreased [369,372,378,379,381,382] after phthalate exposure (Table 2).

##### Perchlorate

Perchlorate is a chemical substance used in the production of propellants, pyrotechnics, airbags and fertilizers and is approved as a food contact substance (therefore, it can be released into various foods, milk and water) [383]. Perchlorate reduces the intake of iodine in the thyroid because it is an inhibitor of NIS [384]. Studies in healthy adults generally observed a decrease in T4 [381,385,386,387,388] and T3 levels [381] after exposure to perchlorate, while TSH levels were either not affected [386,388] or increased [385] (Table 2). Although only a few studies have been conducted so far, they have all included a sufficient number of participants. In addition, a similar pattern of TSH and thyroid hormone levels could be observed among studies: a decrease in thyroid hormone levels, with TSH levels remaining unchanged (Table 2). This indicates that perhaps perchlorate first exerts its effect on thyroid hormones.

##### Perfluoroalkyl Substances

Perfluoroalkyl substances (PFASs) can resist both water and oil and are therefore used as surfactants in products such as textiles, paints, food packaging, cookware and cosmetics [389]. PFASs inhibit the synthesis and increase the metabolic excretion of thyroid hormones [390]. Many studies have tested the effect of PFASs on thyroid hormone homeostasis in healthy adults. The results were inconsistent and showed no effect [254,345,391,392,393,394,395,396,397,398,399,400,401,402,403,404], increase [254,401,405,406] or decrease [344,398] in TSH levels after exposure to PFASs. No effect [254,345,398,399], increase [254,392,395,396,397,402,404,406] or decrease [344,401,406] in T3 levels and no effect [254,393,395,396,397,399,404,406], increase [254,344,345,394,397,400,402] or decrease [392,398,401,405] in T4 levels were observed after exposure to PFASs (Table 2).

##### Pesticides

Pesticides are EDCs, and various in vitro and in vivo studies have shown that pesticides, including insecticides, fungicides and herbicides, alter normal thyroid function (reviewed in [407,408]). Pesticides affect the metabolism and production of thyroid hormones (reviewed in [409]). The effect of various pesticides on TSH and thyroid hormone levels in healthy adults was tested (phenoxybenzoic acid (3-PBA) (metabolite of pyrethroid insecticide), trichloro-2-pyridinol (TCPY) (a metabolite of chlorpyrifos), *cis and trans*-3-2,2-dichlorovinyl-2,2-dimethylcyclopropane carboxylic acid (*cis* and *trans*-DCCA) (pyrethroid metabolites), 1-napththol (1N) (a metabolite of carbaryl and naphthalene), ethylene bisdithiocarbamate (EBDC) fungicides, insecticide fipronil sulfone metabolite, dithiocarbamate fungicides, lambda-cyhalothrin (pyrethroid), paraquat (herbicide), *p*,*p*′-dichlorodiphenyltrichloroethane (DDT), *p*,*p*′-diphenyldichloroethene (DDE), hexachlorobenzene (HCB), alpha-chlordane, endosulphan 2, methoxychlor, beta-hexachlorocyclohexane (HCH) and mancozeb (fungicide)) (Table 2). Some studies have compared TSH and thyroid hormone levels between conventional farmers (who use pesticides) and organic farmers [410]. Studies have yielded inconsistent results. Pesticide use increased [341,410,411,412,413,414,415,416,417], decreased [415,418,419,420] or had no effect [352,355,356,413,421,422,423,424,425,426,427,428,429,430,431,432] on TSH levels. T4 levels either increased [343,410,412,414,415,416,420,433,434,435], decreased [344,410,416,421,422,423,426,427,428,429,433,436] or did not change [341,352,355,356,411,413,417,421,423,424,425,427,428,430] after pesticide use. The same was observed with T3: studies reported an increase [343,410,416,420,433,434,435], decrease [343,344,414,416,417,429,436,437,438] or no change [352,355,413,423,424,426,427,428,430,433] after pesticide use. Such variations in results are expected since different types of pesticides were analyzed.

##### Nitrate

Nitrate can occur naturally in vegetables grown in soil and in surface water and groundwater. However, due to excessive use of fertilizers, septic systems in rural areas, food processing waste and industrial waste, nitrate levels in the food and water can increase. Nitrate ion competitively binds to NIS, resulting in low iodine intake in the thyroid gland (reviewed in [439]). Higher exposure to nitrate has even been associated with a higher risk of developing hypothyroidism [440,441]. Studies examining the effect of nitrate on TSH and thyroid hormone levels in healthy adults have yielded inconsistent results. Some studies have observed a decrease [442], an increase [443] or no change [385] in TSH levels as a result of higher nitrate exposure. Moreover, a decrease [385,387], an increase [442] or no change [443,444,445] in T4 levels was observed as a result of higher nitrate exposure, while T3 levels did not correlate with nitrate levels [444,445].

#### 3.2.2. Heavy Metals

Heavy metals such as arsenic (As), cadmium (Cd), lead (Pb) and mercury (Hg) are environmental toxins that interfere with the normal functioning of the thyroid gland. Arsenic has been shown to inhibit TPO activity [446]. Cadmium affects TPO activity [329,447] and alters thyroid hormone metabolism [329,448]. Lead affects the intake of iodide in the thyroid gland [329] and alters the metabolism of thyroid hormones [449]. Mercury affects TPO activity [329,447] and inhibits deiodinases involved in the metabolism of thyroid hormones [450]. Various studies examining the effect of heavy metals on TSH and thyroid hormone levels in healthy adults have yielded inconsistent results. Arsenic exposure leads to an increase [318,451,452,453], decrease [453] or no change [453,454] in TSH levels. T4 levels decreased [451,453], while T3 levels either decreased [451] or did not change [453] after arsenic exposure. After cadmium exposure, an increase [455], a decrease [456] or no change [318,457,458,459,460,461,462,463] in TSH levels was observed. T4 levels increased [456,457,458,462], decreased [455,463,464] or did not change [423,457,458,459,460,461] after cadmium exposure. The same was observed for T3; an increase [456,457,458,462,464], decrease [455,463] or no change [458,459,460,461] after cadmium exposure. Lead exposure caused an increase [460,461,465,466,467], a decrease [318,351,468] or no change [423,454,457,458,459,469,470,471,472,473,474,475] in TSH levels. Likewise, an increase [458,468,476,477], a decrease [318,458,465,470,473,478] or no change [351,423,457,459,460,461,465,466,469,471,472,474,475,478] in T4 levels and an increase [351,423,458,468,477], a decrease [465,472] or no change [423,457,459,460,461,466,470,471,473,474,475] in T3 levels were observed after lead exposure. TSH levels were increased [479,480] or unchanged [318,423,456,457,481,482] after exposure to mercury. T4 levels were also increased [480,481], decreased [456,457,480,482] or unchanged [351,423,456,457,479] after exposure to mercury. Inconsistent results for T3 were also observed: studies reported an increase [483], a decrease [456,457,481,482] or no change [479] after mercury exposure (Table 2). The cause of variability between the studies was probably due to the fact that the participants were exposed to different doses of heavy metals. Moreover, the majority of conducted studies were underpowered. Therefore, additional studies with a larger number of participants are needed to elucidate the influence of heavy metals on TSH and thyroid hormone levels.

**Table 2:** Effect of pollutants on thyroid-stimulating hormone, thyroid hormone and thyroglobulin levels in healthy individuals.

Factor	Compounds Used in the Study			Effect on Hormone Levels	Number of Participants	Reference
**Chemicals**	Polychlorinated biphenyls and polybrominated biphenyls	PBB		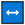 TSH, ↓fT4, ↑fT3, ↑tT3, ↑fT3/fT4	715	[349]
		PCB		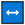 TSH, ↑fT4, 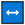 fT3, ↑fT3/fT4		
		PCBs and hydroxylated PCBs		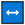 TSH, 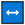 fT4, 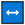 tT4, 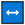 fT3, 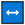 tT3	79	[348]
		PCB		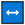 TSH, ↑fT4, ↑tT4, ↑fT3, ↑tT3	551	[347]
		PCB		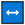 TSH, ↓fT4, ↓fT3	122	[346]
		PCB		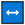 TSH, ↓fT4, ↑tT4, 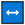 tT3	87	[345]
		PCB		↓TSH, ↑fT4, ↑tT4, ↑fT3, ↑tT3	67	[353]
		PCB		↓tT3	114	[435]
		PCB x BDE		↑tT3		
		PCB		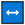 TSH, 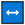 fT4, ↓tT3	623	[344]
		PCB		↑TSH, ↓T4, ↓T3	211	[351]
		PCB		↑TSH, ↓fT4, 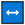 tT4, 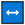 tT3	232	[423]
		PCB		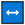 TSH, ↑fT4, ↑tT3	2042	[484]
		PCB		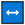 TSH, 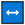 fT4, ↓tT3	341	[343]
		PCB		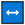 TSH, ↑fT4	2045	[422]
		PCB		↓tT4	2445	[356]
		Dioxin-like toxic equivalents		↑TSH, ↓tT4		
		PCB		↓TSH	454	[418]
		PCB		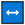 TSH, 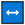 fT4	196	[341]
		PCB		↓TSH, ↓tT4, ↓tT3	66	[352]
		Dioxin-like toxic equivalents		↓TSH		
		PCB		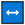 TSH, 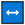 fT4, 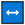 tT4, 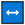 fT3, 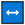 tT3	110	[354]
		PCB		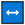 TSH, 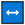 fT4, 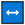 tT4, 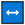 fT3, ↓tT3	182	[339]
		PCB		↓T4	229	[355]
		PCB		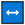 TSH, 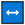 fT4, 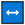 tT4	192	[421]
		PCB		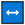 TSH, 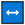 fT4, 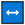 tT4, 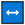 fT3	173	[340]
		PCB		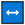 tT4	111	[350]
	Polybrominated diphenyl ethers	PBDE		↑TSH, 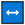 fT4, 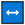 tT4, ↑fT3, ↓tT3	85	[362]
		PBDE		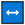 TSH, 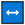 fT4, ↑tT4, 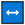 fT3, ↑tT3	79	[348]
		PBDE		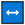 TSH, 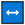 fT4, ↓tT4, 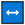 tT3	52	[361]
		PBDE		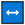 TSH, 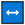 fT4, ↑tT3	623	[344]
		PBDE		↑TSH	49	[363]
		PBDE		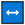 TSH, 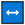 fT4, 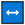 tT4, 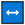 tT3	36	[360]
		PBDE		↓TSH, ↑tT4, ↓tT3, ↑rT3	308	[364]
		PBDE (BDE-47)		↓TSH, 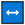 tT4, 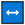 fT4, 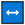 tT3, 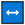 fT3	110	[354]
	Bisphenol A	BPA		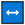 TSH, 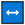 tT4, ↑tT3	90	[368]
		BPA		↓TSH, 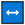 tT4, 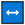 tT3	6003	[372]
		BPA		↑TSH, 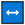 fT4	194	[370]
		BPA		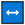 TSH, ↓fT4	2340	[367]
		BPA		↓TSH, 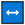 fT4, ↑fT3	3394	[371]
		BPA		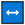 TSH, 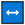 fT4, 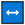 tT4, 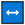 tT3, 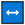 Tg	1346	[369]
		BPA		↓TSH, 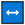 fT4, 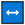 tT3	167	[373]
	Phthalates	DEHP metabolites and MEHHP		↓tT4	Meta-analysis (included studies on pregnant women and children)	[382]
		MEOHP		↓fT4		
		MEHHP, DEHP metabolite		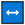 TSH, 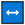 fT4, ↓T4, 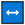 T3	279	[379]
		MEHP, MEOHP		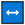 TSH, ↓fT4, 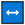 T4, 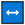 T3		
		Monoethyl phthalate		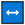 TSH, ↑fT4, 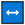 T4, 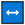 T3		
		MEOHP		↑TSH, 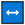 tT4, 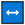 tT3	6003	[372]
		DEHP metabolites		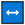 TSH, ↓tT4, 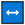 tT3		
		MnBP		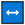 TSH, 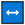 tT4, ↓tT3		
		MnBP		↑TSH, 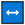 fT4	43 (all subjects)	[380]
		MnBP, 5Cx-MEP, 5Oxo-MEHP, MBzP		↑TSH, 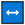 fT4	30 (women)	
		MEHHP		↓tT4	1877 (all subjects)	[381]
		MEOHP		↑tT4	907 (women)	
		DEHP		↑TSH, ↓fT4, ↓tT4, ↓tT3, ↓Tg	1346	[369]
		MEHP		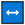 TSH, ↓fT4, ↓tT3	408	[378]
	Perchlorate	Perchlorate		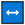 TSH, ↓fT4	2702	[388]
				↓fT4	564	[387]
				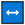 TSH, ↓fT4, ↓tT4	4023	[386]
				↓fT4, ↓tT4, ↓fT3, 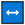 tT3	1877	[381]
				↑TSH, ↓tT4	1111	[385]
	Perfluoroalkyl substances	PFAS		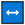 TSH, 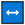 fT4, 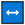 fT3	3297	[399]
		PFOS, PFNA, PFAS, PFHxS		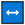 TSH, ↑fT4, 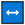 tT4, 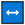 fT3, 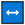 tT3	1325	[400]
		PFOA		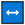 TSH, ↓tT4, ↓fT4, 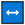 tT3	3070	[398]
		PFOS		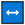 TSH, ↓tT4, 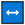 fT4, 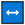 tT3		
		PFNA, PFDeA		↓TSH, 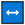 tT4, 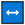 fT4, 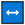 tT3		
		PFOA, PFNA		↑TSH, 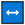 tT4, 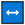 fT4, 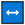 tT3, 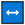 fT3	85	[406]
		PFNA		↑tT3, ↑fT3	47 (women)	
		PFNA		↓tT3, ↓fT3	38 (men)	
		PFOS		↑fT3	47 (women)	
		PFOS		↓fT3	38 (men)	
		PFOS		↑TSH, ↓tT4, ↑fT4, ↓tT3	Meta-analysis (including pregnant women)	[401]
		PFOA		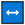 TSH, ↓tT4, 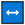 tT3		
		PFNA		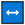 TSH, 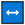 T4, ↑fT4, ↑T3	99	[397]
		PFOA		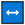 TSH, 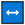 T4, 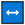 fT4, ↑T3		
		PFOA		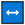 TSH, 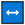 fT4, 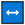 tT4, 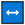 tT3, ↑fT3	1012	[396]
		PFOS		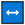 TSH, ↑fT4, ↑tT4, 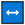 tT3	87	[345]
		PFOS, PFNA		↓TSH, 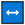 tT4, 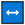 fT4, 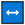 tT3, 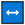 fT3	158 (male adolescents)	[402]
		PFOA		↓TSH, 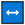 tT4, 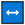 fT4, 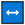 tT3, 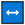 fT3	145 (female adolescents)	
		PFOA, PFOS, PFNA		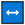 TSH, 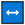 tT4, ↑fT4, 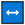 tT3, 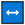 fT3	257 (women 20-40 years old)	
		PFOA		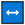 TSH, 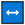 tT4, 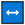 fT4, ↑tT3, ↑fT3	199 (women 60-80 years old)	
		PFNA		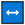 TSH, ↑fT4	567	[403]
		PFOA		↑TSH, 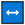 tT4, 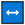 fT4, ↑tT3, 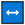 fT3, 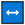 Tg	1540	[254]
		PFHxS		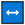 TSH, ↑tT4, 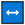 fT4, 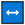 tT3, 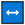 fT3, 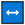 Tg		
		PFOA		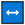 TSH, 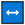 tT4, 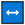 fT4, ↑tT3, 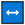 fT3	509 (women)	[395]
		PFHxS		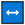 TSH, ↑tT4, 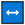 fT4, ↑tT3, 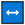 fT3	509 (women)	
				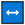 TSH, 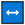 tT4, ↓fT4, 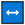 tT3, 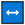 fT3	672 (men)	
		PFTrDA		↑TSH, ↓tT4	633	[405]
		PFOS, PFOA		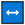 TSH, ↑tT4	50,113	[394]
		PFC		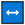 TSH, 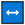 fT4	31	[393]
		PFOS		↓TSH, ↑fT4, ↓tT3	623	[344]
		PFOA		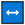 TSH, ↓fT4, 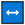 T4, ↑T3	506	[392]
		PFOA		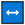 TSH	371	[391]
		PFOS		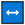 TSH, 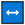 fT4, 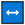 T4, ↑T3	255	[404]
	Pesticides	Conventional farmers that use insecticides, herbicides and fungicides in comparison to organic farmers		↑TSH, ↓fT4, ↑T4, ↑fT3, ↑T3	438	[410]
		Organophosphate insecticides		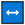 TSH, 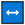 fT4, 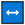 tT4, 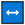 fT3, 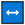 tT3	41	[432]
		Rural workers exposed to pesticides in comparison to controls		↓TSH, ↑fT4, ↑tT3	73	[420]
		3-PBA (metabolite of pyrethroid insecticide)		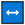 TSH, ↓tT4, ↓tT3	6208	[429]
		Insecticides and pyrethroids for >20 years		↓fT4, ↓tT3	106	[436]
		TCPY (a metabolite of chlorpyrifos)		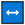 TSH, ↓tT4, 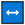 fT4, 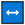 tT3, 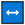 fT3, ↓Tg	2015	[427]
		Mancozeb (fungicide)		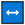 TSH, 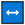 fT4, ↓T4, ↑fT3, 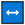 T3, ↓Tg	63	[428]
		3-PBA		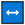 TSH, 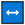 fT4, 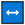 tT4, 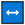 fT3, 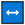 tT3, 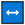 Tg	2015	[430]
		*p*,*p*′-DDE (a stable metabolite of DDT)		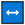 TSH, ↑tT4, ↑tT3	136	[434]
		Pesticide sprayers exposed to organophosphate and organochlorine pesticides		↑TSH, 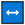 T4, ↓T3	60	[417]
		DDT+DDE		↑tT4, ↑tT3	48 (women)	[435]
		DDT+DDE + PCB		↑tT4		
		DDT+DDE + PCB		↓tT3	66 (men)	
		Exposure to organophosphate and carbamate pesticides		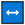 TSH, 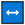 fT4	99	[425]
		High exposure pesticide season		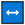 TSH, ↓fT4, 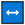 tT3	91	[426]
		HCH		↑TSH, ↓fT4	303 (men)	[416]
		HCB, heptachlor, *o*,*p*′-DDT and *p*,*p*′-DDT		↑fT4	305 (women)	
		Endosulphan 2		↓tT3	303 (men)	
		Alpha-chlordane, *p*,*p*′- DDT, endosulphan 2 and methoxychlor		↑tT3	305 (women)	
		TCPY (a metabolite of chlorpyrifos)		↓TSH, ↑tT4	1589 (men)	[415]
				↑TSH	218 (women)	
		Insecticide fipronil sulfone metabolite		↓TSH, 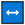 fT4, 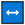 tT4	155	[419]
		DAP		↑TSH, ↑tT4	215	[414]
		DMP		↑TSH, ↑tT4, ↓tT3		
		Organochlorine pesticides		↓tT3	623	[344]
		Hexachlorobenzene		↓fT4		
		cis-DCCA (pyrethroid metabolite)		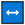 TSH, 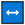 fT4, 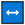 tT3	161	[424]
		3-PBA and trans-DCCA (pyrethroid metabolites)		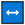 TSH, 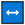 fT4, 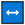 tT3		
		HCB		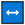 TSH, 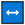 fT4, ↓tT4, 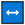 tT3	232	[423]
		DDE		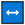 TSH, ↓fT4, ↑tT3	2045	[422]
		HCB		↑TSH, ↓fT4, 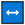 tT3		
		*p*,*p*′-DDE		↓TSH, ↑fT4, ↑tT3	341	[343]
		HCB		↓tT3		
		*p*,*p*′-DDE		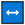 TSH, 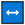 tT4	2445	[356]
		PCB + DDE + HCB		↓TSH	454	[418]
		DDE		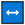 TSH, 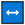 tT4, 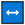 tT3	66	[352]
		*p*,*p*′-DDE		↑TSH, 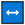 fT4	196	[341]
		High exposure pesticide season		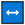 TSH, ↓fT4, 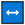 fT3, 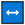 tT3	122	[433]
		In the fall in comparison to the spring season (people are exposed to higher levels of pesticides in fall)		↓TSH, ↑fT4, ↑fT3, ↑tT3		
		TCPY (a metabolite of chlorpyrifos)		↑TSH, 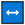 fT4, 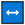 tT3	322	[413]
		1N (a metabolite of carbaryl and naphthalene)		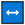 TSH, 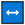 fT4, 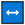 tT3		
		EBDC fungicides		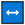 TSH	131	[431]
		HCB		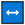 T4	66	[485]
		High exposure pesticide season		↑TSH, ↑fT4, ↑tT4	193	[412]
		DDT, HCB		↓T3	16	[438]
		DDE		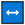 TSH, 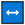 T4, 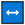 T3	51	[355]
		HCB		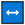 TSH, 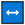 fT4, ↓tT4	192	[421]
		Exposure to organophosphates and organochlorine pesticides		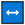 TSH, 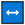 T4, ↓T3	50	[437]
		EBDC fungicides		↑TSH, 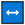 T4	94	[411]
	Nitrate	Nitrate		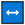 T4, 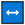 T3	30	[445]
				↑TSH, 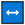 fT4	41	[443]
				↓fT4	307	[387]
				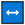 TSH, ↓tT4	1111	[385]
				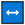 TSH, 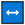 T4, 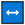 T3	20	[444]
				↓TSH, ↑T4	60	[442]
**Heavy metals**	Studies determining multiple metals	Pb, Cd, As		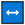 TSH	102	[454]
		Pb		↑TSH, 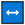 fT4, 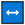 fT3	100	[460]
		Cd		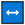 TSH, 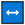 fT4, 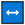 fT3		
		Pb		↑TSH, 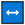 tT4, 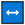 tT3	5628	[461]
		Cd		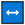 TSH, 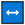 tT4, 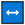 tT3		
		Cd		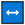 TSH, 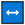 fT4, 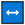 tT4, 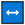 fT3, 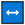 tT3, 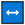 Tg	1391	[459]
		Pb		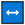 TSH, 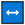 fT4, 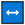 tT4, 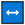 fT3, 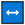 tT3, 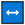 Tg		
		Cd		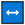 TSH, ↑fT4, 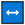 tT4, 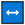 fT3, 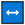 tT3, ↑Tg	6231 (all subjects)	[458]
				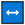 TSH, 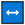 fT4, 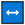 tT4, 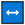 fT3, ↑tT3, ↑Tg	3231 (men)	
		Pb		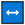 TSH, 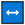 fT4, 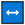 tT4, ↑fT3, 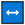 tT3, 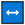 Tg	6231 (all subjects)	
				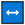 TSH, 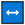 fT4, ↓tT4, 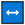 fT3, 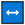 tT3, 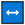 Tg	3231 (men)	
				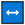 TSH, ↑fT4, 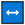 tT4, 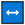 fT3, 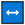 tT3, 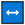 Tg	3000 (women)	
		Hg		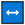 TSH, 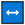 fT4, ↓tT4, ↓fT3, ↓tT3, 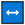 Tg	4409	[457]
		Cd		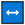 TSH, 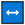 fT4, ↑tT4, ↑fT3, ↑tT3, ↑Tg		
		Pb		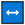 TSH, 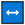 fT4, 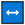 tT4, 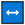 fT3, 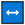 tT3, 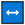 Tg		
		Hg		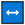 TSH, 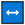 fT4, ↓T4, ↓fT3, ↓T3	1587	[456]
		Cd		↓TSH, ↑fT4, ↑T4, ↑fT3, ↑T3		
		Pb		↓TSH	219	[318]
		As		↑TSH		
		Hg, Cd		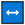 TSH		
		Pb		↓TSH, 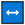 T4, ↑T3	211	[351]
		Hg		↑TSH, 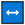 T4, 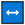 T3		
		Pb		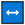 TSH, 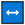 fT4, 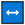 tT4, ↑T3	232	[423]
		Hg		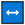 TSH, 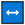 fT4, 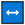 tT4, 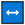 T3		
	Studies determining single metal	Arsenic	UDMA	↑TSH, ↓fT4, ↓tT4, 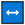 fT3, 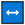 tT3, 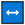 Tg	4126	[453]
			UAAS	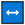 TSH, 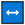 fT4, ↓tT4, 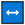 fT3, 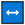 tT3, 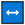 Tg		
			UAS, UAB	↓TSH, 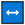 fT4, 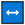 tT4, 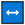 fT3, 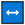 tT3, ↓Tg		
				↑TSH, 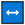 fT4, 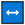 fT3	38	[452]
				↑TSH, ↓fT4, ↓fT3, ↑Tg	185	[451]
		Cadmium		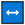 TSH, ↓fT4	1972	[463]
				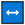 TSH, ↑fT4, ↑fT3	1724	[462]
				↑TSH, ↓fT4, ↓fT3	277	[455]
				↓fT4, ↑tT3	105	[464]
		Mercury		↑TSH, ↑fT4, ↓T4, 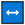 fT3, 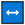 T3	Meta-analysis	[480]
				↑TSH, 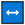 fT4, 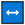 fT3, 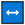 tT3	110	[479]
				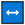 TSH, ↓T4, ↓T3	137	[482]
				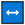 TSH, ↑fT4, ↓fT3, ↑fT4/fT3	82	[481]
				↑rT3, ↑fT4/fT3	94	[483]
		Lead		↓TSH, ↑fT4, ↑fT3	87	[468]
				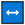 TSH, 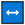 fT4, 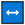 T4, 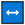 fT3, 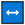 T3	Meta-analysis	[475]
				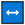 TSH, 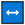 T4, 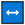 T3	195	[474]
				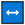 T4, ↓fT4	220	[478]
				↑T3, ↑T4	76	[477]
				↑TSH	125	[467]
				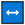 TSH, ↓fT4, 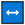 fT3	97	[473]
				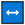 TSH, 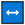 fT4, 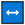 T4, ↓fT3, ↓T3	157	[472]
				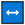 TSH, 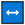 fT4, 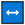 fT3	103	[471]
				↑TSH, 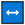 fT4, ↓T4, ↓T3	75	[465]
				↑TSH, 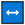 fT4, 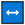 fT3	93	[466]
				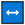 TSH, ↑fT4, ↑tT4, ↑fT3, 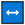 tT3	57	[476]
				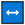 TSH, 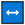 T4	151	[469]
				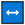 TSH, ↓fT4, ↓tT4, 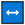 tT3	176	[470]

Studies in pregnant women, infants, kids and individuals with a history of thyroid diseases were not included in this table. 1N, 1-naphthol; 3-PBA, phenoxybenzoic acid; 5Cx-MEP, mono-ethyl phthalate; 5Oxo-MEHP, mono-(2-ethylhexyl) phthalate; As, arsenic; BPA, bisphenol-A; Cd, cadmium; DAP, dialkylphosphate; cis and trans-DCCA, *cis and trans*-3-2,2-dichlorovinyl-2,2-dimethylcyclopropane carboxylic acid; DDE, *p*,*p*′-diphenyldichloroethene; DDT, *p*,*p*′-dichlorodiphenyltrichloroethane; DEHP, di-(2-ethylhexyl) phthalate; DMP, dimethyl metabolites; EBDC, ethylene bisdithiocarbamates; fT3, free triiodothyronine; fT4, free thyroxine; HCB, hexachlorobenzene; HCH, beta-hexachlorocyclohexane; Hg, mercury; MBzP, mono-benzyl phthalate; MEHP, mono(2-ethylhexyl) phthalate; MEHHP, mono (2-ethyl-5-hydroxyhexyl) phthalate; MEOHP, mono-(2-ethyl-5-oxohexyl) phthalate; MnBP, mono-n-butyl phthalate; PBB, polybrominated biphenyls; PBDE, polybrominated diphenyl ethers; PCB, polychlorinated biphenyls; PFAS, perfluoroalkyl substances; PFC, perfluorinated compounds; PFDeA, perfluorodecanoate; PFHxS, perfluorohexane sulfonate; PFNA, perfluorononanoic acid; PFOA, perfluorooctanoic acid; PFOS, perfluorooctane sulfonic acid; PFTrDA; perfluorotridecanoic acid; rT3, reverse triiodothyronine; T3, triiodothyronine; T4, thyroxine; tT3, total T3; tT4, total T4; TCPY, trichloro-2-pyridinol; Tg, thyroglobulin; TSH, thyroid-stimulating hormone; UAAS, arsenic adjusted for arsenobetaine; UAB, arsenobetaine; UAS, total arsenic; UDMA, dimethylarsinic acid.

## 4. Conclusions

The scope of this review was to provide a comprehensive insight into the literature discussing the influence of environmental factors on TSH and thyroid hormone levels in healthy adults. We included lifestyle factors (smoking, alcohol consumption, diet and exercise) and pollutants (chemicals and heavy metals) (Figure 1). After analyzing the literature, we conclude that there is still a large variability in results between studies. The pollutant that showed the clearest relationship with thyroid hormones was perchlorate; most studies have noticed a decrease in thyroid hormone levels after exposure to perchlorate (Table 2). Lifestyle factors that showed the highest consistency in results between studies were smoking, BMI and iodine (micronutrient taken from the diet). Smoking leads to a decrease in TSH levels and an increase in T3 and T4 levels (Table 1). There was a positive correlation between BMI levels and TSH and fT3 levels (Table 1). In addition, an increase in TSH levels and a decrease in thyroid hormone levels were observed after excess iodine (Table 1). Future studies should continue to analyze the influence of environmental factors on thyroid function. Studies should involve a large number of participants and meta-analyses should also be conducted. More studies in this area will provide researchers with valuable information needed to understand the complex background of gene–environment interactions that underlie the development of thyroid disease.

## Data Availability

Data sharing is not applicable. No new data were created or analysed in this study.

## Abbreviations

1N	1-naphthol
3-PBA	phenoxybenzoic acid
5Cx-MEP	mono-ethyl phthalate
5Oxo-MEHP	mono-(2-ethylhexyl) phthalate
As	arsenic
BMI	body mass index
BPA	bisphenol A
Cd	cadmium
DAP	dialkyl phosphate
cis and trans-DCCA	cis and trans-3-2,2-dichlorovinyl-2,2-dimethylcyclopropane carboxylic acid
DDE	*p*,*p*′-diphenyldichloroethene
DDT	*p*,*p*′-dichlorodiphenyltrichloroethane
DEHP	di-(2-ethylhexyl) phthalate
Dio1	type 1 iodothyronine deiodinase
Dio2	type 2 iodothyronine deiodinase
DMP	dimethyl metabolite
EBDC	ethylene bisdithiocarbamate
EDC	endocrine-disrupting compound
ESS	euthyroid sick syndrome
fT3	free triiodothyronine
fT4	free thyroxine
GWAS	genome-wide association studies
HCB	hexachlorobenzene
HCH	beta-hexachlorocyclohexane
Hg	mercury
HPT axis	hypothalamus–pituitary–thyroid axis
MBzP	mono-benzyl phthalate
MEHP	mono(2-ethylhexyl) phthalate
MEHHP	mono (2-ethyl-5-hydroxyhexyl) phthalate
MEOHP	mono-(2-ethyl-5-oxohexyl) phthalate
MnBP	mono-n-butyl phthalate
NIS	sodium/iodide symporter
PBB	polybrominated biphenyl
PBDE	polybrominated diphenyl ether
PCB	polychlorinated biphenyl
PFAS	perfluoroalkyl substance
PFC	perfluorinated compound
PFDeA	perfluorodecanoate
PFHxS	perfluorohexane sulfonate
PFNA	perfluorononanoic acid
PFOA	perfluorooctanoic acid
PFOS	perfluorooctane sulfonic acid
PFTrDA	perfluorotridecanoic acid
POP	persistent organic pollutant
rT3	reverse triiodothyronine
T3	triiodothyronine
T4	thyroxine
tT3	total T3
tT4	total T4
TCPY	trichloro-2-pyridinol
Tg	thyroglobulin
TPO	thyroid peroxidase
TRH	thyrotropin-releasing hormone
TSH	thyroid-stimulating hormone
UAAS	arsenic adjusted for arsenobetaine
UAB	arsenobetaine
UAS	total arsenic
UDMA	dimethylarsinic acid
VDR	vitamin D receptor
